# Recent Advances on Magnetic Sensitive Hydrogels in Tissue Engineering

**DOI:** 10.3389/fchem.2020.00124

**Published:** 2020-03-06

**Authors:** Zhongyang Liu, Jianheng Liu, Xiang Cui, Xing Wang, Licheng Zhang, Peifu Tang

**Affiliations:** ^1^Department of Orthopedics, Chinese PLA General Hospital, Beijing, China; ^2^National Clinical Research Center for Orthopedics, Sports Medicine and Rehabilitation, Beijing, China; ^3^Beijing National Laboratory for Molecular Sciences, Institute of Chemistry, Chinese Academy of Sciences, Beijing, China

**Keywords:** magnetic particle, magnetic hydrogel, magnetic field, tissue engineering, functional recovery

## Abstract

Tissue engineering is a promising strategy for the repair and regeneration of damaged tissues or organs. Biomaterials are one of the most important components in tissue engineering. Recently, magnetic hydrogels, which are fabricated using iron oxide-based particles and different types of hydrogel matrices, are becoming more and more attractive in biomedical applications by taking advantage of their biocompatibility, controlled architectures, and smart response to magnetic field remotely. In this literature review, the aim is to summarize the current development of magnetically sensitive smart hydrogels in tissue engineering, which is of great importance but has not yet been comprehensively viewed.

## Introduction

Tissue engineering, a branch of regenerative medicine, refers to the application of supporting cells, material scaffolds, bioactive molecules, or their combinations to repair and reconstruct tissues and organs. Hydrogels have been shown to be one of the most applicable biomaterials in tissue engineering (Kabu et al., 2015; Madl et al., 2017, 2019; Deng et al., 2019) mainly attributed to their inner 3D network microstructures, moderate biocompatibility, and good water content feature, which are analogous with those of the natural tissue (Cui Z.K. et al., 2019; Zhu et al., 2019). Meanwhile, hydrogel-based drug delivery systems for numerous therapeutic agents, with high water content, low interfacial tension with biological fluids, and soft consistency, have been shown to be more stable, economical, and efficient in comparison with conventional delivery systems (Li and Mooney, 2016; Moore and Hartgerink, 2017; Cheng et al., 2019; Fan et al., 2019; Zheng et al., 2019). Considering the above advantages, hydrogels have been conducted into the biomedical application to provide a tunable three-dimensional scaffold for cell adhesion, migration, and/or differentiation, and they could also be designed as the platform for the controlled release of cytokines and drugs in tissue engineering and drug delivery (Huang et al., 2017; Jiang et al., 2017; Hsu et al., 2019; Wei et al., 2019; Zheng et al., 2019).

Hydrogel first appeared in a literature as early as in 1894 (Van Bemmelen, 1894); however, the “hydrogel” described at that time was not the same form of hydrogels used nowadays; it was a type of a colloidal gel made from inorganic salts. Later on, the term “hydrogel” was applied for describing a 3D network of hydrophilic native polymers and gums by physical or chemical crosslinking approaches, and its application heavily relied on water availability in the environment (Lee et al., 2013). The current generation of hydrogel in the biological field was first performed by Wichterle and LÍm (1960), indicating that glycoldimethacrylate-based hydrophilic gels exhibited adjustable mechanical properties and water content. From then on, more and more hydrogels have been developed, and the smart hydrogels were then introduced in different fields of biological science, such as drug delivery, bioseparation, biosensor, and tissue engineering. Smart hydrogels are described as they respond directly to the changes of environmental conditions (Wichterle and LÍm, 1960), and numerous studies of smart hydrogels in the applications of nanotechnology, drug delivery, and tissue engineering have been put into effect in the last few decades (Li X. et al., 2019; Li Z. et al., 2019; Zhang Y. et al., 2019).

Recently, magnetically responsive hydrogel, as one kind of smart hydrogels, has been introduced into biomedical applications in improving the biological activities of cells, tissues, or organs. This is mainly attributed to its magnetic responsiveness to external magnetic field and obtaining functional structures to remotely regulate physical, biochemical, and mechanical properties of the milieu surrounding the cells, tissues, or organs (Abdeen et al., 2016; Antman-Passig and Shefi, 2016; Rodkate and Rutnakornpituk, 2016; Bannerman et al., 2017; Omidinia-Anarkoli et al., 2017; Xie et al., 2017; Silva et al., 2018; Tay et al., 2018; Wang et al., 2018; Bowser and Moore, 2019; Ceylan et al., 2019; Luo et al., 2019). Recent studies have represented that magnetic hydrogel could act as an excellent drug release and targeting system. For example, Gao et al. (2019) fabricated a magnetic hydrogel based on ferromagnetic vortex-domain iron oxide and suggested that this unique magnetic hydrogel could significantly suppress the local breast tumor recurrences. Manjua et al. (2019) developed magnetic responsive poly(vinyl alcohol) (PVA) hydrogels, which could be motivated by ON/OFF magnetic field and non-invasively regulated protein sorption and motility, indicating a promising application for tissue engineering, drug delivery, or biosensor system. Moreover, a composite magnetic hydrogel prepared by a combination of a self-healing chitosan/alginate hydrogel and magnetic gelatin microspheres could be used as a suitable platform for tissue engineering and drug delivery (Chen X. et al., 2019a). In comparison with magnetic hydrogels, various kinds of smart biomaterials (e.g., scaffolds, biofilms, other smart hydrogels), which are activated by external stimuli, such as light, pH, temperature, stress, or charge, have great potential in biomedical applications (Chen H. et al., 2019; Cui L. et al., 2019; Wu C. et al., 2019; Zhao et al., 2019; Yang et al., 2020). However, the long response time and less precisely controlled architectures of these stimuli-responsive smart biomaterials are the two main limitations.

Magnetic hydrogels are usually made of a matrix hydrogel and a magnetic component that was incorporated into the matrix. Recently, superparamagnetic and biocompatible iron oxide-based magnetic nanoparticles (MNPs) are most commonly incorporated into polymer matrices to prepare magnetically responsive hydrogels for their application in tissue engineering, such as γ-Fe_2_O_3_, Fe_3_O_4_, and cobalt ferrite nanoparticles (CoFe_2_O_4_) (Zhang and Song, 2016; Rose et al., 2017; Ceylan et al., 2019). Magnetite (Fe_3_O_4_) is a compound of two kinds of iron sites with 1/3 of Fe^2+^ and 2/3 of Fe^3+^. The intervalence charge transfer between Fe^2+^ and Fe^3+^ induces absorption throughout the ultraviolet–visible spectral region and the infrared spectral region, which generates a black appearance in color (Barrow et al., 2017). Maghemite (γ-Fe_2_O_3_), with a brown-orange color pattern, is an oxidative product of magnetite (Fe_3_O_4_) when the temperature is below 200°C (Tang et al., 2003). In terms of CoFe_2_O_4_, previous studies have shown that the concentration of 20% was toxic, whereas at 10% the toxicity was insignificant. Moreover, 10% (w/w) of CoFe_2_O_4_ could maximize magnetic response because of numerous amounts of nanoparticles, developing biocompatible biomaterials (Goncalves et al., 2015; Brito-Pereira et al., 2018). For example, Hermenegildo et al. (2019) designed a novel CoFe_2_O_4_/Methacrylated Gellan Gum/poly(vinylidene fluoride) hydrogel, which created a promising microenvironment for tissue stimulation.

In this literature review, we aim to summarize the preparation methods and current development of magnetically sensitive smart hydrogels in tissue engineering, especially in bone, cartilage, and neural tissue engineering, which are of great importance but have not yet been comprehensively reviewed.

## The Fabrication Processing of Magnetic Hydrogels

**Figure 1:**
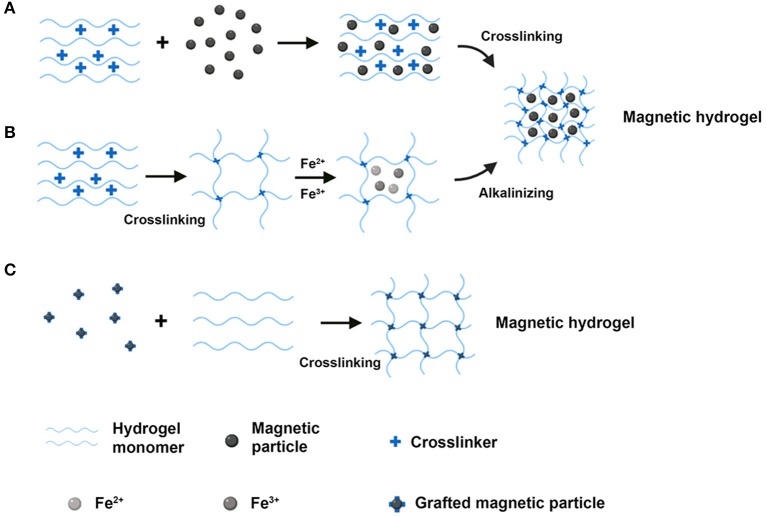
The schematic diagram of the main three routes for the synthesis of the magnetic hydrogels. **(A)** The blending method. **(B)** The *in situ* precipitation method. **(C)** The grafting-onto method (Created with BioRender.com).

Magnetic hydrogels are made of composite materials that possess biocompatibility, biodegradation, and magnetic responsiveness. The characteristics of magnetic hydrogels depend upon several issues, including the magnetic particles and the component of hydrogels used, the magnetic particles and hydrogels' concentration, and the size and uniformity of magnetic particles within the hydrogels. There are mainly three preparation methods of fabricating magnetic hydrogels: (**i**) blending method; (**ii**) *in situ* precipitation method; (**iii**) grafting-onto method (Table 1). The scheme for the main three syntheses of the magnetic hydrogels is shown in Figure 1.

**Table 1:** The preparation methods of magnetic hydrogels and the parameters of magnetic field in representative references.

**Method**	**Magnetic particles**	**Hydrogels**	**Magnetic particle concentration**	**Physiochemical properties**	**Magnetic field**	**References**
Blending method	Fe_3_O_4_	Bisphosphonate-modified HA	2 w/v%	Proper rheology and fast heat-generation	Alternating magnetic field	Shi et al., 2019
	Fe_3_O_4_	Chitosan/PEG	0–40 wt%	Nanoheat	Alternating magnetic field	Cao et al., 2018
	Fe_3_O_4_	NIPAAM-MAA	2.5 mg/mL	Uniform distribution of particles	–	Namdari and Eatemadi, 2017
	Fe_2_O_3_	Poly(vinyl alcohol)/n-HAP	4 wt%	High water content and good elasticity	–	Huang J. et al., 2018
	Magnetite	Hyaluronate hydrogel	0.2, 2.0 g/L	Stable and homogeneous dispersion in 3 months	–	Tóth et al., 2015
	Magnetite	Collagen	0.5 mg/mL	Aligned collagen fibers and normal electrical activity	Magnet (255 G)	Antman-Passig and Shefi, 2016
	Dextran iron oxide composite particles (Micromod®)	Agarose	20 wt% (surface) 7 wt% (middle) 10 wt% (deep)	Gradients in compressive modulus	Rare earth NdFeB magnets (0.4, 0.5, or 0.75 T; E-Magnets®)	Brady et al., 2017
	Streptavidin-coated magnetic particles	Agarose/collagen	10 v/v%	Mimicking the native multilayered tissues	Magnet (2 mT)	Betsch et al., 2018
	Nano-HAP-coated γ-Fe_2_O_3_ nanoparticles (m-nHAP)	Poly(vinyl alcohol)	0–80 wt%	Linearly saturated magnetic strength and porous structures, homogenous dispersion of m-nHAP and improved compressive strength	–	Hou et al., 2013
	PEG-functionalized iron oxide (II, III) nanoparticles	PEG hydrogel (modified with factor XIIIa)	1 mg/mL	Smooth inner gel texture, slow relaxation kinetics, and high elastic modulus	Neodymium magnets (50 mT)	Filippi et al., 2019
	MNPs	Collagen	–	Bio-mimetic 3D structures	Static magnetic fields	Yuan et al., 2018
	MNPs	Poly(lactide-co-glycolide)	1, 5, and 10 wt%	Homogenous distribution of MNPs and linear structures	Standard cuvette Magnets (100–300 mT)	Omidinia-Anarkoli et al., 2017
	MNPs	Six-arm star-PEG-acrylate	0.0046 vol%	Unidirectional structures and high controlled properties	Magnets (100, 130, and 300 mT)	Rose et al., 2017
	MNPs	RGD peptides modified alginate	7 wt%	Fatigue resistance	Magnet (6,510 G, 1 Hz)	Cezar et al., 2016
	MNPs	GRGDSPC peptides/six-arm PEG-acrylate	400 μg/mL	Tailed properties	Magnet (150 mT)	Rose et al., 2018
*In situ* precipitation method	Fe_3_O_4_	Chitosan	0–15 wt%	Uniform distribution of MNPs and enhanced mechanical properties	Low frequency magnetic field (60 Hz)	Wang et al., 2018
	Polydopamine-chelated carbon nanotube-Fe_3_O_4_	Acrylamide	0–15 wt%	Directional conductive and mechanical properties	Low static magnetic field (30 mT)	Liu et al., 2019
	Dextran-coated Fe_3_O_4_	Bacterial cellulose	25–100 mM	Magnetization saturation (10 emu/g) and moderate Young's modulus (200–380 KPa)	Neodymium magnets (0.3 T)	Arias et al., 2018
Grafting-onto method	CoFe_2_O_4_	Polyacrylamide	–	High stability and homogeneity	–	Messing et al., 2011
	Poly(vinyl alcohol) modified Fe_3_O_4_	Hybrid hydrogel (containing HA, collagen, and PEG)	4 wt%	Increased surface roughness and biodegradation	Magnet	Zhang et al., 2015
	3-(trimethoxysilyl)propyl methacrylate coated Fe_3_O_4_	Polyacrylamide	20–60% (with respect to the total weight of the hydrogels and water)	High mechanical properties and excellent underwater performance (polydimethylsiloxane coating)	–	Hu et al., 2019
	Carboxyl-coated Nanomag® superparamagnetic nanoparticles (Micromod®)	RGD-tripeptide; TREK1-antibody	–	–	Mica Biosystem bioreactor	Henstock et al., 2014
	Saline modified carbonyl iron particles	Polyacrylamide	–	Elastic hysteresis	Alternating magnetic field	Abdeen et al., 2016
	Glycosylated MNPs	Agarose	10^11^ glycosylated MNPs in 100 μL 1 wt% agarose hydrogel	MNPs-gradient magnetic hydrogel	Finite element magnetic modeling	Li et al., 2018
	Methacrylated chondroitin sulfate (MA-CS)-MNPs	MA-CS enriched with platelet lysate	200 and 400 μg/mL	Homogenous trabecular structures and high storage modulus	Oscillating magnet array system	Silva et al., 2018
	Kartogenin (KGN) grafted ultrasmall superparamagnetic iron oxide	Cellulose nanocrystal/dextran hydrogel	0.06–0.3 wt%	Good mechanical strength, long-term sustained KGN release, and stable MRI capabilities	–	Yang et al., 2019
	PEG-magnetic microparticles	Thiolated HA	–	Uniform distribution of MMPs and mimicking the native tissue ECM	Applied magnetic field (2 T)	Tay et al., 2018

### The Blending Method

In the blending approach, magnetic particles, and hydrogels are fabricated separately. The magnetic particles are normally synthesized using a coprecipitation process. The resulting magnetic particles are then stored in an aqueous or oily liquid to prevent aggregation and oxidization. Finally, the solution of magnetic particles is mixed with the hydrogel solution for crosslinking, and the magnetic particles are encapsulated into the hydrogels. Tóth et al. (2015) modified the surface of MNPs (magnetite) with biocompatible chondroitin-sulfate-A (CSA) to develop CSA-coated MNPs. Then, CSA-coated MNPs were dispersed into hyaluronate (HyA) hydrogel to prepare the HyA-based magnetic hydrogel. It was found that the HyA-based magnetic hydrogel could be conducted in the treatment of osteoarthritis by the intra-articular injection approach. Shi et al. (2019) fabricated bisphosphonate (BP)-modified hyaluronic acid (HA)/Fe_3_O_4_ magnetic hydrogel. This novel magnetic hydrogel showed outstanding compatibility and slow degradation *in vivo* and held the expectation of safety in clinic usage.

The blending method has several advantages in the preparation of magnetic hydrogels. Firstly, magnetic particles (e.g., MNPs, micrometer-iron oxides) with homogenous size in hydrogels can be obtained by modifying the stirring speed, the concentration of the substances, and the fabrication period. Secondly, the preparation process is easy to be performed since the preparation and crosslinking of magnetic particles are conducted separately. However, there are also some limitations that could be addressed in the future, including the asymmetric distribution of magnetic particles within the hydrogels and the diffusion of magnetic particles when the magnetic hydrogels were submerged in a solution.

### *In situ* Precipitation Method

In the process of the *in situ* precipitation, the network of the hydrogels acts as a chemically reactive substance, within which the iron ions from inorganic salts in hydrogels react with alkali solutions (e.g., NH_3_·H_2_O, NaOH; Arias et al., 2018; Liu et al., 2019) to prepare the magnetic particles. In detail, the hydrogels are firstly prepared through polymerization, temperature change, or a crosslinking reaction. Then, magnetite precursors containing iron(III) and iron(II) at a molar ratio of 1:2 are added into the hydrogels to obtain a homogenous solution. Finally, the mixture is immersed in an alkali solution to induce the crystallization of magnetite. The magnetic particles are incorporated into the hydrogels according to the following hydrolytic reaction (2 Fe^3+^ + Fe^2+^ + OH– → Fe_3_O_4_ + 4 H_2_O). Wang et al. (2018) designed a magnetic chitosan hydrogel by adding MNPs via *in situ* synthesis during chitosan hydrogel formation. The resulting magnetic hydrogel showed a magnetic response and improved morphological and mechanical features, including the homogenous distribution of MNPs and excellent wettability. Moreover, the mechanical properties (such as the compression strength, the yield strength, and the probe displacement) of the magnetic hydrogel was enhanced with the rising concentration of MNPs from 0 to 15 wt%, which was attributed to the crosslinking role of MNPs in the process. Under low-frequency alternating magnetic field (LAMF) exposure, the magnetic hydrogel laden with drugs exhibited a pulsatile drug release profile. In addition, the *in situ* fabricated MNPs in the magnetic hydrogel had excellent biocompatibility and no acute toxicity on MG-63 cells (human osteosarcoma cell line). Liu et al. (2019) fabricated polydopamine (PDA)-chelated carbon nanotube (CNT)-Fe_3_O_4_ (PFeCNT) nanohybrids into the acrylamide hydrogel via an *in situ* precipitation method to generate the PFeCNT hydrogel. This magnetic hydrogel formed an anisotropic morphology under a low static magnetic field (SMF) (30 mT) and showed conductive and mechanical features. Interestingly, under external electrical stimulation, the myoblasts cultured on the magnetic hydrogel exhibited oriented outgrowth. Arias et al. (2018) designed a magnetic bacterial cellulose (MBC) hydrogel by an *in situ* synthesis approach that mixed the dextran-coated Fe_3_O_4_ nanoparticles with bacterial cellulose pellicles. The resulting MBC hydrogel could be activated with saturation magnetization and exhibited a moderate Young's modulus (200−380 kPa), which was appropriate for vascular tissue engineering. Under an external magnetic field (0.3 T) stimulation, the produced gradient magnetic fields resulted in higher cell retention for the magnetically activated MBC hydrogel.

With the *in situ* precipitation method, numerous kinds of inorganic substances could be applied in the fabrication of the hydrogel networks, with good dispersion in the hydrogel matrix. Moreover, the synthesized approach is straightforward and economical. However, this method is only appropriate for limited hydrogels that possess stable networks. This is because the alkali solution used in the process might destroy the hydrogel network and limit the application of cell encapsulation (Wang et al., 2009).

### The Grafting-Onto Method

In the grafting-onto methodology, there are covalent bonds formed between the magnetic particles and the hydrogel network. In detail, several functional groups are grafted onto the surface of the magnetic particles, which act as micro- or nanocrosslinkers to generate covalent bonds with the hydrogel monomers. Hu et al. (2019) designed a magnetic hydrogel made from non-toxic polyacrylamide (PAAm) hydrogel and 3-(trimethoxysilyl)propyl methacrylate coated Fe_3_O_4_ via the grafting-onto approach. This novel magnetic hydrogel exhibited relatively high mechanical properties, including tensile strength and fracture toughness. In addition, polydimethylsiloxane (PDMS) was introduced to modify the surface of the magnetic hydrogel, and the resulting product exhibited excellent underwater performance. The PDMS-coated magnetic hydrogel kept stability even under fatigue loading, which highlighted significant potentials in the applications of artificial muscles. Messing et al. (2011) firstly fabricated methacrylic groups functionalized magnetic CoFe_2_O_4_ nanoparticles. Then the magnetic hydrogels were fabricated by adding this kind of magnetic nanoparticles into a polyacrylamide hydrogel network.

An advantage of the grafting-onto approach is that the covalent bonds are capable of encapsulating the magnetic particles within the hydrogels, which promote the stability of the magnetic hydrogel. However, the long fabrication time, the high-cost protocol, and the complicated fabrication process restrict its broader applications in the biomedical field.

## Applications in Bone Tissue Engineering

The repair of large bone defects caused by traumas, infections, cancers, or other diseases is still a thorny clinical problem (Herberg et al., 2019). Previous studies have shown that osteo-inductive growth factors played a significant role in bone regeneration (Cui L. et al., 2019; Ruehle et al., 2019) however, autologous bone graft remains the gold standard in the treatment of bone defects (Bouyer et al., 2016). Considering the donor site morbidity and limited source of the donor area, the development of bone tissue engineering biomaterials is becoming more and more attractive for researchers. Most endeavors have been applied for the evaluation of MNPs-incorporated hydrogels in bone repair. As hydroxyapatite (HAP) is one of the most important components in natural bone inorganic substances, exhibits excellent biocompatibility and osteo-conductivity, and plays a key role in biomineralization (Moncion et al., 2019; Zhou et al., 2019), the magnetic HAP composite hydrogel was designed. For example, nano-HAP coated γ-Fe_2_O_3_ nanoparticles (m-nHAP) were synthesized and then added into poly(vinyl alcohol) (PVA) solution to fabricate m-nHAP/PVA hydrogels. The PVA showed excellent biocompatibility, high mechanical properties, and slow biodegradation, which were crucial for its application (Iqbal et al., 2019; Venkataprasanna et al., 2019). The pore sizes of hydrogels rose gradually followed by the increased content of m-nHAP, which was accessible for nutrient exchange. Meanwhile, the adhesion and proliferation of human osteoblasts were dramatically promoted as the m-nHAP concentration increased (Hou et al., 2013). However, the magnetic field was not introduced in this study, which might be considered as a potential promoter in improving bone tissue regeneration using this magnetic hydrogel.

**Figure 2:**
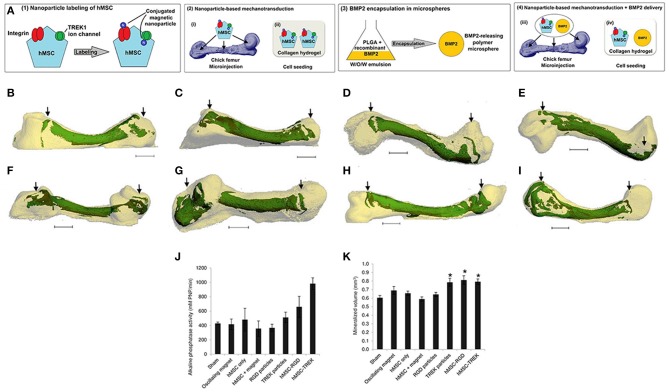
The enhancement of hMSCs prelabeled with either RGD-modified or TREK-antibody-modified MNPs in hydrogel for biomineralization. **(A)** Schematic illustration of the experiment. (A1) The integrins or the TREK1 ion channel modified MNPs were used to label hMSCs. (A2) The MNP-labeled hMSCs were either transplanted into an *ex vivo* chick femur model (A2i) or incorporated into a hydrogel (A2ii). (A3) BMP-2-releasing PLGA microspheres were fabricated using an emulsion method. (A4) The combined effect of labeled cells and BMP-2-releasing hydrogels was studied. **(B–E)** Bone mineralization sites (green) within the cartilaginous chick fetal femur (white) were used to express the location and extent of mineralization. The experiments were conducted as follows: sham injection **(B)**, magnetic stimulation alone **(C)**, injection of hMSCs alone **(D)**, and injection of hMSCs followed by magnetic stimulation **(E)**. **(F,G)** The RGD tripeptide **(F)** or TREK-antibody **(G)** modified MNPs led to mineralization. **(H,I)** The injection of hMSCs prelabeled with either RGD-modified **(H)** or TREK-antibody-modified **(I)**. MNPs in femurs showed more alkaline phosphatase activity **(J)** and the greatest extent of mineralization **(K)**. *p < 0.05. Scale bar = 1 mm [reproduced with permission from Henstock et al. (2014), Copyright 2014 John Wiley and Sons].

Cells, such as stem cells, neural cells, and osteoblasts, are an important component in tissue engineering (Cerqueira et al., 2018; He et al., 2018; Ahmad et al., 2019; Chen M. et al., 2019; Midgley et al., 2019); therefore, MNP labeled cells are becoming more and more available in magnetically bioinspired hydrogels. Henstock et al. (2014) first added TREK1-MNPs or Arg-Gly-Asp (RGD)-MNPs into human mesenchymal stem cells (hMSCs) to fabricate MNP labeled cells, which were then seeded into either collagen hydrogels or poly(D, L-lactide-coglycolide) (PLGA) encapsulating bone morphogenetic protein-2 (BMP-2)-releasing collagen hydrogels (as shown in Figure 2A). The RGD-binding sites of cell-surface mechanoreceptors (i.e., integrins) play an important role in transferring the external stimulus into the intracellular cytoskeleton (Cartmell et al., 2002). It was worth mentioning that hydrogels containing TREK1 or RGD-MNP labeled hMSCs showed significantly more mineralization than controls; moreover, hydrogels containing functionalized MNPs and BMP-2 had thicker and more numerous mineralized domains compared to those without BMP-2 (Figures 2B–K). In order to maintain the stem cell phenotype and multiple differentiation potential *ex vivo*, a magnetic cell levitation method was used to label the MSCs with MNPs and then fabricated multicellular spheroids, which were implanted into type I collagen to form a magnetic hydrogel. Biological analyses showed that the 3D spheroid hydrogel system retained the functionality of MSCs, maintained the expression of stem cell markers, produced hematopoietic factors, and decreased cell-cycle progression genes, which could be able to establish an attractive platform for osteogenesis and drug delivery (Lewis et al., 2017).

Critical size bone defects need adequate vascularization for reconstruction (Genova et al., 2016). Therefore, Filippi et al. (2019) designed cell-loading magnetic nanocomposite hydrogels by incorporation of human adipose tissue derived stromal vascular fraction (SVF) cells into polyethylene glycol (PEG)-MNPs-based PEG hydrogels, which were further examined to enhance the activity of alkaline phosphatase (ALP), to increase the expression of osteogenesis-related genes, and to improve the mineralized extracellular matrix (ECM) formation both *in vitro* and *in vivo*. The results suggested that magnetically actuated cell-laden hydrogels demonstrated more mineralization and faster vascularization in comparison with MNPs or SMF alone. In addition, the *in vitro* and *in vivo* tissue volume examinations showed that this magnetic hydrogel was biodegradable. Further studies are needed to investigate the effect of this construct in an orthotopic bone defect model and in combination with other hard supporting materials (e.g., HAP). Although a previous review has focused on the multifunction of magnetic HAP in nanomedicine application (Mondal et al., 2017), the characteristics involved in the magnetic PEG hydrogels should be tested comprehensively.

Previous studies have shown that the cell activity in the local milieu relies on the spatiotemporal regulation of biophysical and biochemical properties (Lukashev and Werb, 1998), and the ECM plays an important role in this process (Chantre et al., 2019; Ebata et al., 2019; Park et al., 2019; Zhang Z. et al., 2019). The introduction of the ECM into the preparation of hydrogels could mimic the microenvironment for cell behaviors, which is mainly attributed to predetermined hydrogel properties, such as the ultrastructure (Johnson et al., 2011, 2014), stiffness (Massensini et al., 2015; Ghuman et al., 2016), gelation kinetics (Medberry et al., 2013; Wu et al., 2015; Lin et al., 2018), etc. Therefore, the development of mimicked matrices is imperative for the regulation of ECM characteristics that control cellular biology. Abdeen et al. (2016) designed a magnetoactive hydrogel matrix fabricated by incorporating carbonyl iron particles into a polyacrylamide (PAAM) hydrogel, and the elasticity was modulated reversibly by alternating magnetic field from −1.0 to 1.0 T. It was worth mentioning that the storage modulus of the magnetic hydrogel had shown elastic hysteresis. A conditioned medium from MSCs seeded on magnetic hydrogels promoted the tube formation of functional vessels, which provides nutrients, oxygen, and ions for biomineralization (Qiu et al., 2020), suggesting enhanced secretion of proangiogenic factors from MSC loaded hydrogels. Runx2, the most common biomarker as the early osteogenesis, was significantly enhanced when MSCs were seeded on the magnetic hydrogel at day 10.

**Figure 3:**
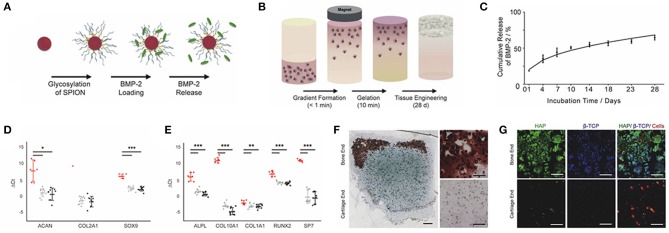
The improvement of aligned glycosylated MNPs within an hMSC-laden agarose hydrogel in the osteochondral tissue engineering. **(A)** MNPs were incorporated into heparin to develop a glycosylated group. **(B)** Aligned glycosylated MNPs within an hMSC-laden agarose hydrogel was fabricated by an external magnetic field. **(C)** The release of BMP-2 from the magnetic agarose hydrogel was analyzed over 28 days. ΔCt values for the bone region (black), the cartilage region (gray), and a day 0 control (red) for genes associated with **(D)** cartilage formation and **(E)** bone formation (mean ± 95% confidence intervals, *N* = 3, *n* = 3 for 28 day constructs. *N* = 3, *n* = 1 for day 0 control; the *COL2A1* gene was only detected in one donor on day 0, but was detected for all 28-day samples). **(F)** Calcium nodules (red) stained by Alizarin Red S and sulfated glycosaminoglycan (blue) stained by Alcian Blue dying indicated mineralization at the bone end. **(G)** Roman microscopy of the cells (red), HAP (green), and β-TCP (blue) suggested mineralization at the bone end. Scale bar = 100 μm. *p < 0.05, **p < 0.01, and ***p < 0.001 [reproduced with permission from Li et al. (2018), Copyright 2018 Elsevier].

It is reported that the structural, compositional, and functional complexity of the native interface is present between bone and tendon (Phillips et al., 2008; Seidi et al., 2011), which is particularly mechanosensitive (Klein-Nulend et al., 2005; Chen et al., 2010). Therefore, a magnetic responsive hydrogel containing methacrylated chondroitin sulfate (MA-CS), platelet lysate (PL), and MNPs was fabricated. The preparation of MA-CS MNPs showed a superparamagnetic feature at room temperature and provided methacrylic groups for crosslinking to the hydrogel matrix. Human adipose-derived stem cells (hADSCs) or human tendon-derived cells (hTDCs) were encapsulated into hydrogels, and biological performance was evaluated. It was worth mentioning that the hydrogel possessed a gradient-like 3D structure with two interconnected layers (bone and tendon) and exhibited non-degradation without magnetic field during the testing period (20 days). Both hTDCs and hADSCs were viable within this construct. Interestingly, under magnetic field stimulation, the magnetic hydrogel showed degradation, and the cellular response of hADSCs on bone-mimicking units and hTDCs on tendon-mimicking units was modulated (Silva et al., 2018). Later on, a new gradient material was designed by incorporating continuous gradients of BMP-2 loaded glycosylated MNPs into agarose hydrogels with an external magnetic field via the grafting-onto method. Then hMSCs were included in this MNP-gradient magnetic hydrogel (schematic illustration is shown in Figures 3A,B). After a 28-day culture *in vitro*, the cumulative release of BMP-2 in hydrogels showed sustained diffusion-driven release kinetics (Figure 3C); osteogenic genes and chondrogenic genes were both significantly increased in comparison with those at day 0 (Figures 3D,E). Moreover, dense calcium-rich nodes (Figure 3F) and two distinct calcium phosphate types (HAP and β-tricalcium phosphate, β-TCP) (Figure 3G) were observed in the bone region of the hydrogel, suggesting that more mineralization could be produced in this new MNP-gradient hydrogel and providing a novel platform for osteochondral tissue engineering (Li et al., 2018). However, this work was performed *in vitro*, and the overall mechanical properties and the biodegradation rate of this magnetic hydrogel should be tested *in vivo* in the future.

In order to evaluate various osteoblastic functions such as collagen formation, cell adhesion, and osteocalcin production, MG-63 as an experimental model was conducted by Yuan et al. (2018). In this literature, a multifunctional biomimetic 3D magnetic hydrogel was fabricated by incorporating MG-63 cells and MNPs into native collagen hydrogels. In the presence of SMFs, the proliferation, ALP formation, and mineralization of MG-63 cells were significantly enhanced; moreover, the osteogenic related gene expression of *Runx2, BMP-2*, and *BMP-4* was also increased, indicating that this 3D magnetic hydrogel was a biocompatible platform for osteogenesis.

Due to the promotion of blood supply, regulation of bone metabolism, improvement of osteogenesis, and formation of new bone by appropriate temperature stimulation (Zanchetta and Bogado, 2001; Chen et al., 2013), appropriate hyperthermia on magnetic hydrogels highlights potential treatment for bone regeneration. Cao et al. (2018) designed a magnetic hydrogel containing MNPs and chitosan/PEG hydrogel, which showed excellent biocompatibility. It was worth mentioning that the cell viability of MSCs was decreased when the temperature was from 37 to 46°C; moreover, the MSCs could survive at below 46°C, and the growth and morphology had no significant changes. Intriguingly, when the temperature was at 43°C, the MSCs within magnetic hydrogel exhibited good cell viability and the highest ALP activity, which stands for the osteogenic differentiation capability. These findings indicate that magnetic hyperthermia hydrogel could be considered as a potential therapeutic strategy for bone regeneration after bone cancer excision.

## Applications in Cartilage Tissue Engineering

**Figure 4:**
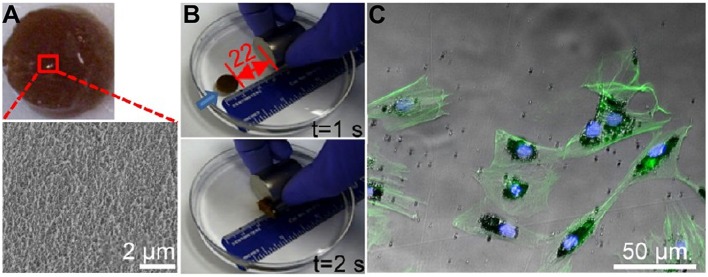
The characteristics of the magnetic hydrogel. **(A)** General view of the magnetic hydrogel. The magnified image was obtained by a scanning electron microscope (SEM). **(B)** Magnetic responses of the magnetic hydrogel. Blue arrows suggested the hydrogel movement orientation before the next time point. **(C)** Representative images of F-actin (green) by fluorescence microscopy and magnetic nanoparticles (black) by light microscopy showed the endocytosis of MNPs by BMSCs [reproduced with permission from Zhang et al. (2015), Copyright 2015 American Chemical Society].

Articular cartilage injury is one of the most usual types of orthopedic disease in the clinic and is challenging for surgeons because of the limitation of self-repair for articular cartilage. Recent endeavors of magnetic hydrogels have been developed to deal with the above issue. A previous study of magnetic hydrogels for potential cartilage tissue engineering was performed by Zhang et al. (2015). In this study, PVA modified Fe_3_O_4_ MNPs were first synthesized via the grafting-on method and then mixed with a hybrid hydrogel (MagGel) composed of HA, type II collagen, and PEG by mechanical dispersion. The *in vitro* degradation test showed that this MagGel lost structure integrity after 21 days' incubation at 37°C. It was worth mentioning that magnetic nanocomposite hydrogel showed similar microstructure and chemical components in comparison with natural hyaline cartilage and was able to support bone mesenchymal stem cells' (BMSCs) behavior *in vitro*, as shown in Figure 4. However, the combination of magnetic hydrogel and magnetic stimulation on cell functions was not evaluated, and the efficacy of magnetic hydrogels on cartilage tissue engineering *in vivo* should be further analyzed. Moreover, HA or chondroitin sulfate (CS), the two important biomolecules of polysaccharides, was used to modify the above-mentioned HAP/Fe_2_O_3_/PVA hydrogel and demonstrated the promotion of chondrocyte attachment and proliferation (Hou et al., 2015).

Later on, an advanced smart cell-embedded magnetic hydrogel was designed. The commercially available, biocompatible, and dextran-modified MNPs from Micromod® company were integrated into a tri-layered agarose scaffold to fabricate a ferrogel by using a blending method. The resulting magnetic hydrogel exhibited a depth-dependent mechanics and was capable of improving cell viability and sustaining the chondrocytes in culture upon magnetic stimulation (Brady et al., 2017). Furthermore, a multilayered mimicked tissue with mimetic architecture consisted of streptavidin-coated magnetic particles (diameter between 10 and 12 nm), agarose, and type I collagen was printed upon a magnetic field by using 3D bioprinting. The new magnetic hydrogel showed similar architecture to cartilage native tissues in comparison to a conventional, single-layered 3D matrix and expressed markedly more chondrocyte-related gene *in vitro* (Betsch et al., 2018). These novel magnetic hydrogels highlighted the potential application of location-specific cartilage replacement tissue, and further studies could be needed to assess the concentration of MNPs and the matrix, the intensity and type of magnetic fields, modification of MNPs with biological growth factors, and the repair efficacy of the damaged cartilage *in vivo*.

The stem cell therapy has been introduced into the treatment of cartilage injury (Toh et al., 2014; Hu et al., 2017); however, the chondrogenic differentiation of BMSCs requires the addition of an important cytokine. The currently available cytokines, such as transforming growth factor-β3 and BMP, have the limitation of short half-life time (Stowers et al., 2013) and less mineralization (Ren et al., 2016), respectively. Kartogenin (KGN) is a small molecule compound and is capable of inducing BMSC differentiation into chondrocytes (Xu et al., 2015). Therefore, KGN was grafted onto the surface of MNPs and then mixed with cellulose nanocrystal/dextran (CNC/Dex) hydrogel. The *in vitro* and *in vivo* experiments showed that KGN-MNPs incorporated hydrogel exhibited a long-term sustained release of KGN, recruited host cells, and induced chondrogenic differentiation of BMSCs, thus improving *in situ* cartilage regeneration (Yang et al., 2019).

**Figure 5:**
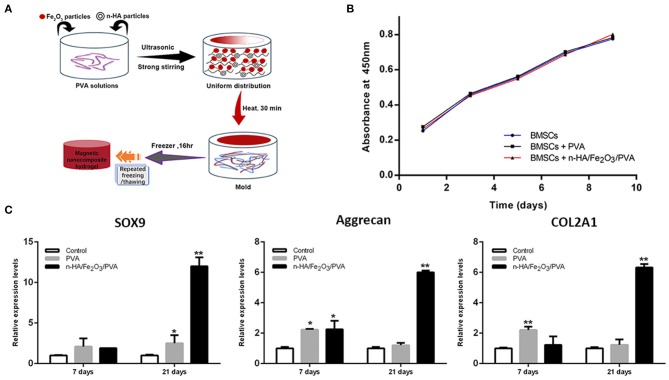
The cell viability and the increased chondrogenesis-related genes in BMSCs within the magnetic hydrogel. **(A)** Schematic diagram of the fabrication of the magnetic hydrogel with nano-HAP particles, Fe_2_O_3_ nanoparticles, and PVA in a ratio of 1:0.5:10. **(B)** Cell viability of BMSCs alone, BMSCs inside PVA hydrogel, and BMSCs within magnetic hydrogel was analyzed by the CCK-8 experiment. **(C)** Chondrogenesis-related genes, such as *SOX9, Aggrecan*, and *COL2A1*, were analyzed by quantitative real-time polymerase chain reaction (PCR). Data were presented as means ± standard deviations. **p < 0.01 and *p < 0.05 vs. control (BMSCs cultured in medium) [reproduced with permission from Huang J. et al. (2018), Copyright 2018 American Chemical Society].

Due to the high biocompatibility, strong toughness, and cell adhesive ability of nano-HAP, many kinds of literatures have reported that nano-HAP possessed effective repair capability for articular cartilage (Reddi et al., 2011; Zhou et al., 2015; Zhu et al., 2017). A magnetic nanocomposite hydrogel made of nano-HAP particles, Fe_2_O_3_ nanoparticles, and PVA in a ratio of 1:0.5:10 was fabricated by using the ultrasonic dispersion approach and then lyophilized by a freeze–thawing crosslinking process, as shown in Figure 5A. Thanks to the Fe_2_O_3_ nanoparticles within the hydrogel, the degradation experiment showed a relatively intermediate and slow mass loss rate. Moreover, the viability of BMSCs in the magnetic hydrogel had no significant change compared to BMSCs alone (Figure 5B), and the expression of chondrocyte-related genes was significantly stimulated, as shown in Figure 5C (Huang J. et al., 2018).

## Applications in Neural Tissue Engineering

Neural tissue damages caused by either neurodegenerative diseases or high-energy trauma greatly affect the patients' quality of life worldwide. The mammalian neuronal cells show the limitation of regrowth performance and functional recovery, presenting a critical clinical challenge for surgeons (Yi et al., 2018; Zhang P.-X. et al., 2019). Most endeavors have been conducted to create a modified microenvironment for improving neural regeneration by supportive tissues and scaffolds. It has been reported that directly guiding the regenerated neurite outgrowth is able to promote neuronal regeneration and functional recovery (Huang L. et al., 2018; Shahriari et al., 2019). A hydrogel matrix containing MNPs and collagen hydrogel was designed, and then an external magnetic field was used to control the alignment of the collagen fibers remotely. After 7-day culture, neurons within a 3D aligned magnetic hydrogel complex exhibited good cell viability and more elongations and more directionality in comparison with those of without fiber alignment, indicating that 3D aligned magnetic hydrogels open up a promising possibility for directional neuronal regeneration (Antman-Passig and Shefi, 2016).

**Figure 6:**
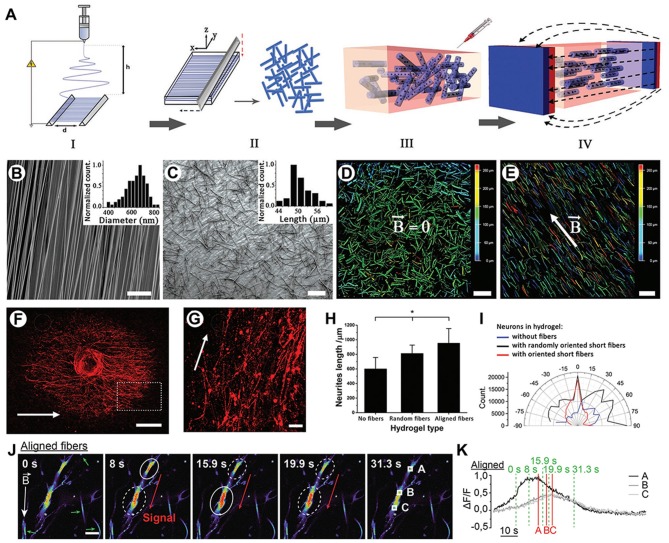
The preparation of the Anisogels and its effect on DRG neurites. **(A)** Schematic illustration of the Anisogel preparation: step I, aligned fibers were electrospun on a parallel plate; step II, the resulting fibers were rinsed with distilled water to wash off redundant gels; step III, random short fibers were added into the hydrogel precursor; step IV, Anisogels were obtained by applying an external magnetic field. **(B)** Representative images of aligned PLGA fibers by SEM. Scale bar = 50 μm. **(C)** The average diameter of 50 μm in short fibers was observed by SEM. Scale bar = 50 μm. Representative images of magnetic fibers within hydrogels by a microscope without magnetic field exposure **(D)** and with 100 mT of magnetic field exposure **(E)**. Scale bars = 100 μm. **(F)** DRG extension in Anisogels. Scale bar = 500 μm. **(G)** A magnified image of DRG extension in Anisogels. Scale bar = 100 μm. **(H)** The length of neurite extensions was quantified. **(I)** The angular distribution of neurite extensions of single neuron. **(J)** Neurons were cultured within the aligned magnetic hydrogel. Red arrows indicated the calcium signal direction, while green arrows represented fibers. A solid circle indicated a maintained or increasing signal. **(K)** Normalized quantification of the calcium signals in the aligned magnetic hydrogel. *p < 0.05 [reproduced with permission from Omidinia-Anarkoli et al. (2017), Copyright 2017 John Wiley and Sons].

In order to rebuild the anisotropic architectures of the ECM, which support and instruct cells for neural regeneration, most aligned nanofibers with appropriate physical and biological properties have been tailored using electrospinning (Li et al., 2004; Corey et al., 2007; Ginestra, 2019; Hou et al., 2019; Idini et al., 2019; Hazeri et al., 2020). Omidinia-Anarkoli et al. (2017) developed a simple but effective strategy to create a novel “Anisogel” containing short, magnetically inspired poly(lactide-co-glycolide) (PLGA) fibers with evenly distributed elements by using a high-throughput electrospinning/micro-cutting approach, as shown in Figures 6A–E. Nerve cells attached well and extended unidirectionally within the “Anisogel” compared to that without fibers or oriented fibers, as shown in Figures 6F–I. Meanwhile, the neurons within the aligned magnetic hydrogel exhibited spontaneous electrical potential by triggering calcium signals, as shown in Figures 6J,K. Later on, magnetic responsive poly-L-lactic acid (PLLA) fibers were prepared by electrospinning and then injecting into a collagen or fibrinogen hydrogel solution. The resulting alignment of MNPs-PLLA fibers was obtained using an external magnetic field. This aligned electrospun fibers within hydrogels provided directional guidance to neurons, and the average length of neurites from dorsal root ganglions (DRGs) on magnetic fibers was significantly longer than those without MNP addition.

Due to the type, severity, and post-injury time of trauma, an injectable hydrogel matrix with minimal invasion and suitable elasticity for the regeneration of various damaged tissues has been developed (Yu and Ding, 2008; Macaya and Spector, 2012; Cheng et al., 2013; Li et al., 2014, 2015). Therefore, the rod-shaped MNP-incorporated microgels were formed, then dispersed in a biocompatible gel precursor, organized unidirectional alignments under a weak external magnetic field exposure, and finally developed a magnetoresponsive hydrogel “Anisogel.” Primary DRGs inside the Anisogels with 3 vol% microgels revealed a clear difference in the orientation of neurite outgrowth in comparison with those random microgels. In addition, fibroblasts cultured on this Anisogel showed no significant difference in cytotoxicity compared with that cultured in a medium (Rose et al., 2017). These findings were performed *in vitro*; therefore, further studies are needed to be conducted *in vivo* to evaluate the efficacy of neurite regeneration and functional recovery *in vivo* by the injection of the magnetic hydrogel system.

**Figure 7:**
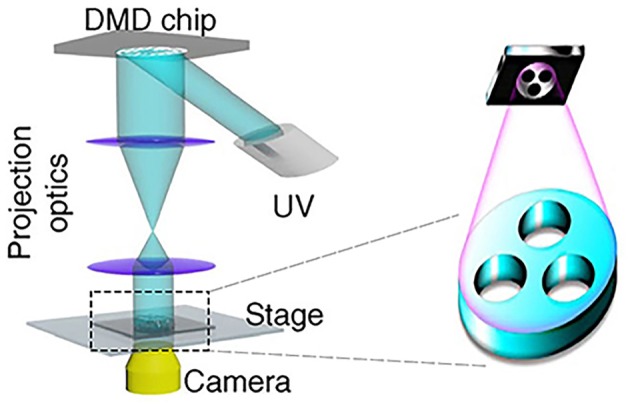
Schematic diagram of 3D printing by a microscale continuous projection printing method [reproduced with permission from Koffler et al. (2019), Copyright 2019 Springer Nature].

Due to the similar biophysical and biochemical features of HA to the natural ECM of the spinal cord and brain (Bignami et al., 1993), a 3D magnetic HA hydrogel was synthesized by the magnetic microparticles, 4-arm-PEG-vinylsulfone, and thiolated HA. The resulting hydrogels had similar storage modulus as the spinal cord, and primary DRG neurons inside the magnetic hydrogels with magnetic fields showed healthy morphology, with no significant difference in cell viability compared to other groups. Interestingly, the calcium influx in DRG neurons was activated via mechanosensitive TRPV4 and PIEZO_2_ channels under acute magnetic exposure, and the expression of PIEZO_2_ was reduced when DRG neurons were stimulated by chronic magnetic exposure. This tailored magnetic biomaterial provided a general strategy for the regulation of different types of cells under remote magnetic stimulation (Tay et al., 2018). Moreover, a recent study has demonstrated that a biomimetic spinal cord structure was printed by a microscale continuous projection printing method (Figure 7), and this hydrogel loaded with neural progenitor cells was capable of promoting axon regeneration in a complete spinal cord injury model *in vivo* (Koffler et al., 2019), providing a promising strategy in the design of novel magnetic hydrogels in the neural tissue engineering.

## Applications in Other Organs

Besides the bone, cartilage, and nerve organs, magnetic hydrogels are also introduced into other organs, such as the heart, skin, and muscle, in order to evaluate the therapeutic potential. Namdari and Eatemadi (2017) designed a magnetic hydrogel by dissolving Fe_3_O_4_ and curcumin into the N-isopropylacrylamide-methacrylic acid (NIPAAM-MAA) hydrogel. The resulting magnetic hydrogel nanocomposite was able to reduce the doxorubicin-induced cardiac toxicity and hold the cardioprotective capability. Cezar et al. (2016) fabricated a ferrogel scaffold by using RGD peptides modified alginate and iron oxide. The magnetic ferrogel showed fatigue resistance and in combination with magnetic stimulation (6,510 Gauss, 5 min at 1 Hz every 12 h) could mechanically activate and promote severely injured muscle tissue regeneration. Rose et al. (2018) developed a magnetic hybrid hydrogel by blending the MNPs into the Gly-Arg-Gly-Asp-Ser-Pro-Cys (GRGDSPC) modified six-arm-PEG gel for fibroblast alignment, which is crucial for the wound healing.

## Injectable Magnetic Hydrogel's Application in Tissue Engineering

Recently, the magnetic hydrogels as injectable systems have displayed great potential for tissue repair and magnetic drug targeting. Various kinds of cells and molecules can be encapsulated homogeneously into the magnetic hydrogels and then targeted to the pathological sites with minimal invasiveness (Wu et al., 2018; Chen X. et al., 2019b; Shi et al., 2019; Wu H. et al., 2019; Xu et al., 2019). Several polymers, including ionic-response polymers (e.g., sodium alginate), natural biocompatible polymers (e.g., chitosan), and synthetic polymers (e.g., polyacrylic acid), conjugated with the magnetic particles have been used to fabricate the injectable hydrogels for therapeutic applications (Jalili et al., 2017; Hu et al., 2018; Amini-Fazl et al., 2019). These magnetic hydrogels could be guided to the diseased sites via external magnetic fields and exert a drug release influence there. Meanwhile, this kind of magnetic hydrogel is mainly targeted for the cancer therapy due to the hyperthermia effect under the applied magnetic field.

## The Metabolism of Magnetic Particles From the Magnetic Biomaterials

Although numerous studies have shown that magnetic biomaterials exhibited biocompatibility both *in vitro* and *in vivo*, the cytotoxicity and long-term fate of magnetic particles embedded within the magnetic hydrogels *in vivo* must be taken for consideration. No identical criteria have been made to evaluate this important issue of magnetic hydrogels since the fabrication process and physicochemical properties vary in many aspects. The Food and Drug Administration (FDA) in the United States had approved several MNPs in the clinical applications, such as the treatment of anemia caused by chronic kidney disease and the magnetic resonance imaging, and these MNPs could be removed quickly by the liver (Laconte et al., 2005; Ventola, 2017). The clearance of magnetic particles *in vivo* depends on the size of the particle. In detail, particles smaller than 5.5 nm could be removed quickly through the kidney (Sun et al., 2008), particles up to 200 nm could be sequestered by phagocytes of the spleen (Chen and Weiss, 1973), and particles larger than 5 μm could be cleared via the lymphatic system (Arruebo et al., 2007). In addition, magnetic fields have proven to control the anisotropic feature, constitution, and the degradation rate in the magnetic hydrogels (Huang J. et al., 2018; Silva et al., 2018). All these factors are crucial for the released amounts of magnetic particles from the magnetic constructs and the impact on organs. More efforts are needed to develop more controllable magnetic hydrogels for *in vivo* applications.

## Conclusions and Perspectives

**Figure 8:**
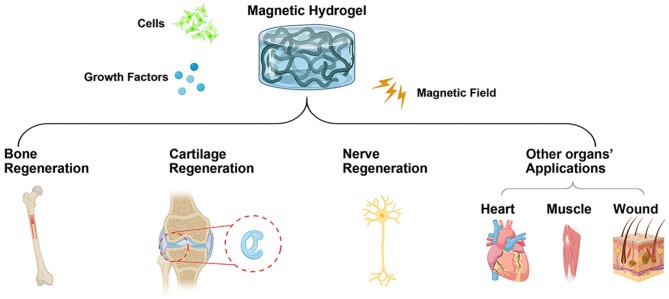
Schematic illustration of combined strategies for the development of magnetic hydrogels in tissue engineering (Created with BioRender.com).

The magnetically responsive smart hydrogels have emerged as an immensely potential biomaterial for developing bioaligned actuators. Benefiting from their intriguing features, including but not limited to quick response, mimetic native tissues, appealing mechanical properties, and biocompatibility, magnetic hydrogels have undergone unparalleled advances in biomedical fields, such as bone, cartilage, nerve, heart, muscle tissue engineering, and so on. However, the properties of magnetic particles, such as size, shape, composition, crystallinity, and so on, should be further modified in order to avoid overheating when cell-laden hydrogels are assembled. Meanwhile, the hydrogel matrices used in magnetic hydrogels need to be further evaluated by mimicking the native architectures of tissues and organs, which holds great potential for the preclinical treatment. Therefore, combined strategies should be developed to create more and more smart hydrogels in tissue engineering (Figure 8).

Future perspectives should focus on dealing with the existing difficulties. In addition, more attention should be taken into consideration in evaluating the magnetic hydrogels' pharmacokinetics/toxicokinetics, metabolism, biodegradation *in vivo*, and so on, which are of great significance in the applications of tissue engineering.

## Author Contributions

PT and ZL initiated the project. ZL, JL, XC, XW, LZ, and PT searched the data and wrote, revised, and completed the manuscript.

### Conflict of Interest

The authors declare that the research was conducted in the absence of any commercial or financial relationships that could be construed as a potential conflict of interest.
